# Distinct expression profiles of peptides in placentae from preeclampsia and normal pregnancies

**DOI:** 10.1038/s41598-020-74840-0

**Published:** 2020-10-16

**Authors:** Jin Huang, Zhonghui Ling, Hong Zhong, Yadong Yin, Yating Qian, Mingming Gao, Hongjuan Ding, Qing Cheng, Ruizhe Jia

**Affiliations:** 1 Women’s Hospital of Nanjing Medical University, Nanjing Maternity and Child Health Care Hospital, Nanjing, 210004 Jiangsu China; 2 Yixing People’s Hospital, YiXing, 214200 Jiangsu China; 3 Fourth Clinical Medicine College, Nanjing Medical University, Nanjing, 210000 Jiangsu China

**Keywords:** Diseases, Health care, Medical research

## Abstract

This study sought to identify potential bioactive peptides from the placenta that are involved in preeclampsia (PE) to obtain information about the prediction, diagnosis and treatment of PE. The liquid chromatography/mass spectrometry was used to perform a comparative analysis of placental peptides in normal and PE pregnancies. Gene ontology (GO), pathway analysis and ingenuity pathway analysis (IPA) were used to evaluate the underlying biological function of the differential peptides based on their protein precursors. Transwell assays and qPCR were used to study the effect of the identified bioactive peptides on the function of HTR-8/SVneo cells. A total of 392 upregulated peptides and 420 downregulated peptides were identified (absolute fold change ≥ 2 and adjusted *P* value < 0.05). The GO analysis, pathway analysis, and IPA revealed that these differentially expressed peptides play a role in PE. In addition, the up-regulated peptide “DQSATALHFLGRVANPLSTA” derived from Angiotensinogen exhibited effect on the invasiveness of HTR-8/SVneo cells. The current preliminary research not only provides a new research direction for studying the pathogenesis of PE, but also brings new insights for the prediction, diagnosis and treatment of PE.

## Introduction

Preeclampsia (PE) is a pregnancy complication characterized by abrupt hypertension and signs of maternal dysfunction after 20 weeks of gestation, and is the leading cause of maternal and perinatal morbidity and mortality^1,2^. PE puts mothers at risk for complications such as gestational diabetes, cardiovascular dysfunction, and chronic renal failure^3–5^. Even after giving birth, a significant proportion of patients have postpartum conditions such as cardiomyopathy and long-term kidney disease^3,6–8^. Considering the risks associated with PE, there is an urgent need to discover the causal mechanisms of PE so that effective predictive and therapeutic interventions can be developed.

Accumulating evidence suggests that the placenta has always been a core factor in the etiology of PE, and pathological examination of placentae from pregnant women with preeclampsia in late pregnancy often reveals numerous placental infarcts and sclerotic narrowing of arterioles^9^. Despite decades of research, the underlying mechanism of pathological placenta in the onset and progression of PE is largely unknown. By recent efforts, obstetric practices for PE have been established in the case of severe maternal disease characteristics or fetal compromise, timely and rapid control of hypertension, restriction of fluids, prevention of high-risk seizures, and accelerated delivery are core principles^10,11^. However, in the clinic, effective treatments for PE are still lacking.

Peptidomics, a new branch of proteomics, has recently received widespread attention^12,13^. Peptides, important biologically active molecules that consist of 3–50 amino acids, are involved in almost all physiological activities^14,15^. Due to the fast development of natural peptide purification and mass spectrometry techniques, the peptidome is becoming increasingly important for identifying biomarkers and therapeutic targets^16–19^. Peptides related to PE have also become a research hotspot in recent years. Some studies have screened peptides from the sera and amniotic fluid of patients with PE for relevant investigation and some meaningful findings have been obtained. For example, the circulating peptide ligand ELABELA (ELA) in serum was found to play an important role in the development of placental vasculature, and at the same time, regulate the changes in the circulatory system during maternal delivery^20^. However, although there have been no direct studies on the role of placenta in PE, during pregnancy, abnormal placental function makes the most sense in relation to the occurrence of PE. Therefore, an in-depth analysis is worthwhile to identify differentially expressed placental peptides that are conducive to the diagnosis and treatment of PE, which perhaps may help clinicians predict, diagnose, and treat PE.

In this study, we compared the peptides of placentae derived from pregnant women with PE and healthy pregnant women. We quantified the differentially expressed peptides by liquid chromatography–tandem mass spectrometry (LC–MS/MS) with tandem mass tag (TMT) labeling. Gene ontology (GO) analysis, canonical pathway analysis and ingenuity pathway analysis (IPA) of the identified differential peptides were also performed. We further found that an up-regulated peptide "DQSATALHFLGRVANPLSTA" derived from Angiotensinogen (AGT) cold increase the trophoblast cells invasion though transwell assay. We aimed to identify potential bioactive peptides involved in PE and help develop promising regimens for prediction, diagnosis and treatment of PE.

## Material and methods

Specimen collection and preprocessing: This study was conducted in accordance with the Declaration of Helsinki and approved by the Human Research Ethics Committee of the Women's Hospital of Nanjing Medical University. The participants were fully informed regarding the aims and scope of the study and willingly signed informed consent forms. According to 2015 ACOG Clinical Guide, patients with severe preeclampsia were diagnosed as one or more of the following: (1) blood pressure ≥ 160/110 mmHg, (2) proteinuria ≥ 5000 mg/24 h, (3) headache or visual impairment, (4) abnormal liver function, (5) right upper quadrant pain, (6) platelet ≤ 100 × 10^9/L. Exclusion criteria for participation include multiple gestation, chronic hypertension, serious infections, placental abnormalities (e.g. underweight, obesity or abnormal invasion) as well as renal or liver disease. Pregnant women with alcohol, drug abuse or habit of smoking were also excluded from this study.

Three placenta samples were separately collected from healthy women and pregnancies with severe preeclampsia at 30–39 weeks of gestation by cesarean section. All samples were obtained immediately after delivery and the tissue block was dissected from the umbilical cord root (about 5 cm from the umbilical cord insertion position) on the maternal surface of the placenta. Each placenta was examined prior to sampling, the diaphragm, the basal plate and the area containing calcification or infarction were identified and excluded. All placental samples were rinsed with sterile phosphate-buffered saline to remove maternal blood from the tissue surface. Tissues were snap frozen and stored at − 80 °C. The entire process was carried out on ice and the samples were processed within 30 min of delivery and sterile practices were strictly observed during the sampling process.

Peptide extraction and desalting: Placental samples were cut into pieces in liquid nitrogen and placed in buffer containing 1 nM PMSF, 2 mM EDTA and 10 mM DTT, followed by ultrasonication of the homogenate for 10 min on ice. Samples were centrifuged at 120,000×* g* at 4 °C for 30 min to obtain supernatants. The protein concentration was determined by the Bradford method. Ultrafiltration was carried out using a 10 KD ultrafiltration tube (centrifugation at 12,000 rpm for 30 min at 4 °C), and then desalted by vacuum freeze-drying on a C18 column to obtain peptides^21^.

iTRAQ labeling and separating component: The peptide was solubilized with 0.5 M TEAB and labeled according to the iTRAQ-8 standard kit (SCIEX) instructions, followed by mixing the labeled peptides and using an Ultimate 3000 HPLC system (Thermo DINOEX, USA) for fractionation. Finally, a total of 42 samples were collected and combined into 12 fractions; then the fractions were desalted on a Strata-X column and dried under vacuum.

LC–MS/MS analysis: LC–MS/MS is a common method to identify peptides or low molecular weight proteins based on its detection sensitivity and throughput capabilities. We chose the Triple TOF 5600plus LC/MS system for the acquisition of mass spectrometry and used a triple TOF 5600plus mass spectrometer coupled to an EKsigent nanoLC system (SCIEX USA) to perform analysis. The polypeptide solution was applied to a C18 trap column (5 µm, 100 µm × 20 mm) and subjected to a gradient elution on a C18 analytical column (3 µm, 75 µm × 140 mm) with a 90 min time gradient at a flow rate of 300 nL/min. The two mobile phases were buffer A (2% acetonitrile/0.1% formic acid/98% H_2_O) and buffer B (98% acetonitrile/0.1% formic acid/2% H_2_O). For information-dependent acquisition, a first-order mass spectrum was scanned with an ion accumulation time of 250 ms, and a secondary mass spectrum of 30 precursor ions was acquired with an ion accumulation time of 50 ms. The MS1 spectrum was acquired in the range of 350–1500 m/z while the MS2 was acquired in the range of 100–1500 m/z.

Peptide identification and quantification: All MS/MS spectra of the extractions were searched by using Proteinpilot V4.5, which is a search engine for AB Sciex 5600 plus. For the identified proteins, we confirmed that the unused score ≥1.3 (the confidence level is above 95%) and the proteins containing at least one unique peptide per protein are reliable proteins. For peptide identification and protein quantification, we filtered with CI  ≥  95, and the confident peptides were considered to be more than 95% for protein quantification. We used the iTRAQ labeling method to relative quantifications of samples simultaneously by Proteinpilot software. When the absolute difference reached  ≥  2 times (absolute fold change  ≥  2) and the adjusted *P* value was ≤ 0.05, it was regarded as a significantly altered peptide segment between the two placenta samples.

Bioinformatics analysis: The Blast2go software was used to perform the GO analysis. A pathway analysis was performed to investigate the significant pathways of progenitor proteins of the significant different peptides, following the Kyoto Encyclopedia of Genes and Genomes (KEGG; http:// www.genome.jp/kegg). The online tool PI/MW (https://web.expasy.org/compute.pi/) was applied to calculate the isoelectric point (pI) and the molecular weight (MW). The cleavage sites of the amino acids were determined by using the Biotools software (Bruker, Bremen, Germany). The mass spectrometry proteomics data have been deposited to the ProteomeXchange Consortium (https://proteomecentral.proteomexchange.org) via the iProX partner repository with the dataset identifier PXD016030^22^.

Cell culture and treatments: HTR-8/SVneo (ATCC, American) cells are commonly used to investigate the behavior of gestational trophoblasts. HTR-8/SVneo cells were cultured in basic RPMI Medium 1640 (Gibco by Thermo Fisher Scientific) supplemented with 8% fetal bovine serum (FBS). Cultured cells were maintained at 37 °C with 5% CO_2_ in a humidified environment. The peptides were purchased from Science Peptide Biological Technology Co. Ltd (Shanghai, China). Specifically, peptide was synthesized by a solid phase method, and then purified by high pressure liquid chromatography. The quality of synthesized peptide was assessed by MS analysis.

RNA preparation and quantitative PCR (qPCR): Total RNA was isolated with Trizol reagent (Ambion by Life Technologies). We used traditional methods to extract and purify total RNA. A NanoDrop 2000 spectrophotometer (Thermo Fisher Scientific, Waltham, MA, USA) was used to evaluate the concentration and purity of the isolated RNA. A total of 2 μg RNA from each sample was reverse-transcribed into cDNA with a high-capacity cDNA reverse transcription kit (Thermo Fisher Scientific Baltics USB). A QuantStudio 7 Flex Real-Time PCR system (Applied Biosystems) was used to carry out qPCR. The sequence of primers used for the qPCR was listed in Supplemental Table S1. The expression levels of mRNAs were calculated by the 2-ΔΔCT method and results were normalized to the expression of *GAPDH* and presented as the fold change of each gene. Table 1 shows the primers used for RT-PCR.

**Table 1:** Comparison of characteristics between PE and normal pregnancies.

Clinical features	PE (n = 3)	Normal pregnancy (n = 3)
Age (years)	25.67 ± 2.51	30.33 ± 5.85
Number of pregnancies	1.67 ± 0.58	1.67 ± 0.58
BMI (kg/m^2^)	27.76 ± 3.66	29.39 ± 2.01
Gestation week	33.07 ± 2.60	38.43 ± 0.05
Systolic pressure (mmHg)	144.33 ± 12.85	113.33 ± 5.77
Diastolic pressure (mmHg)	101.67 ± 7.23^a^	74.00 ± 4.58
Proteinuria level (mg/24 h)	8436.07 ± 3192.05a	N/A
Mode of delivery CS (%)	100	100
Birth weight (g)	1960 ± 814.06	3110.00 ± 226.05
Number of female fetuses	2	3
Apgar score	9.67 ± 814.06	10.00 ± 0.00

*PE* preeclampsia; *BMI* body mass index; *CS* cesarean section; *N/A* not applicable.

^a^*P* < 0.05 when compared with normal pregnancy group.

Transwell assays: Invasiveness of the HTR-8/SVneo cells treated with 10 μm peptide were investigated in a Transwell chamber (Millipore, America) containing Matrigel (BD, Biosciences, America). Cells in the upper chamber were incubated in FBS-free RPMI-1640 medium and the lower chamber was filled with RPMI-1640 containing 8% FBS. After 48 h in a 37 °C, 5% CO_2_ incubator, cells that crossed the membrane were fixed with 4% paraformaldehyde and stained by 0.5–1% crystal violet. Chambers were imaged under light microscopy. Each experiment was performed in triplicate.

Statistical analysis: A Student’s *t* test in Statistical Program for Social Sciences (SPSS) v.22 and GraphPad Prism 5.0 was used for statistical comparisons between two groups (n = 3) and for multiple comparisons. Significance was set at *P* value < 0.05 and all data are shown as the means ± standard deviations (SDs). The Benjamini–Hochberg (BH) procedure was used for controlling the False Discovery Rate (FDR) in screening results and significance was set at adjusted *P* value < 0.05 and absolute fold change ≥ 2.

## Results

### Characteristics of the study population

Table 1 shows the basic information about the subjects in the study. All the recruited women were singleton pregnancies and delivered by caesarean section at the appropriate time. Newborns were healthy and there were no cases of asphyxiation. Age, gestational age, body mass index (BMI), count of pregnancies, birth weight, and systolic pressure between the two groups were not significantly different. As expected, there were differences in diastolic blood pressure and proteinuria levels between the two groups (*P* < 0.05).

### Differentially expressed peptides between the PE and normal groups

**Figure 1:**
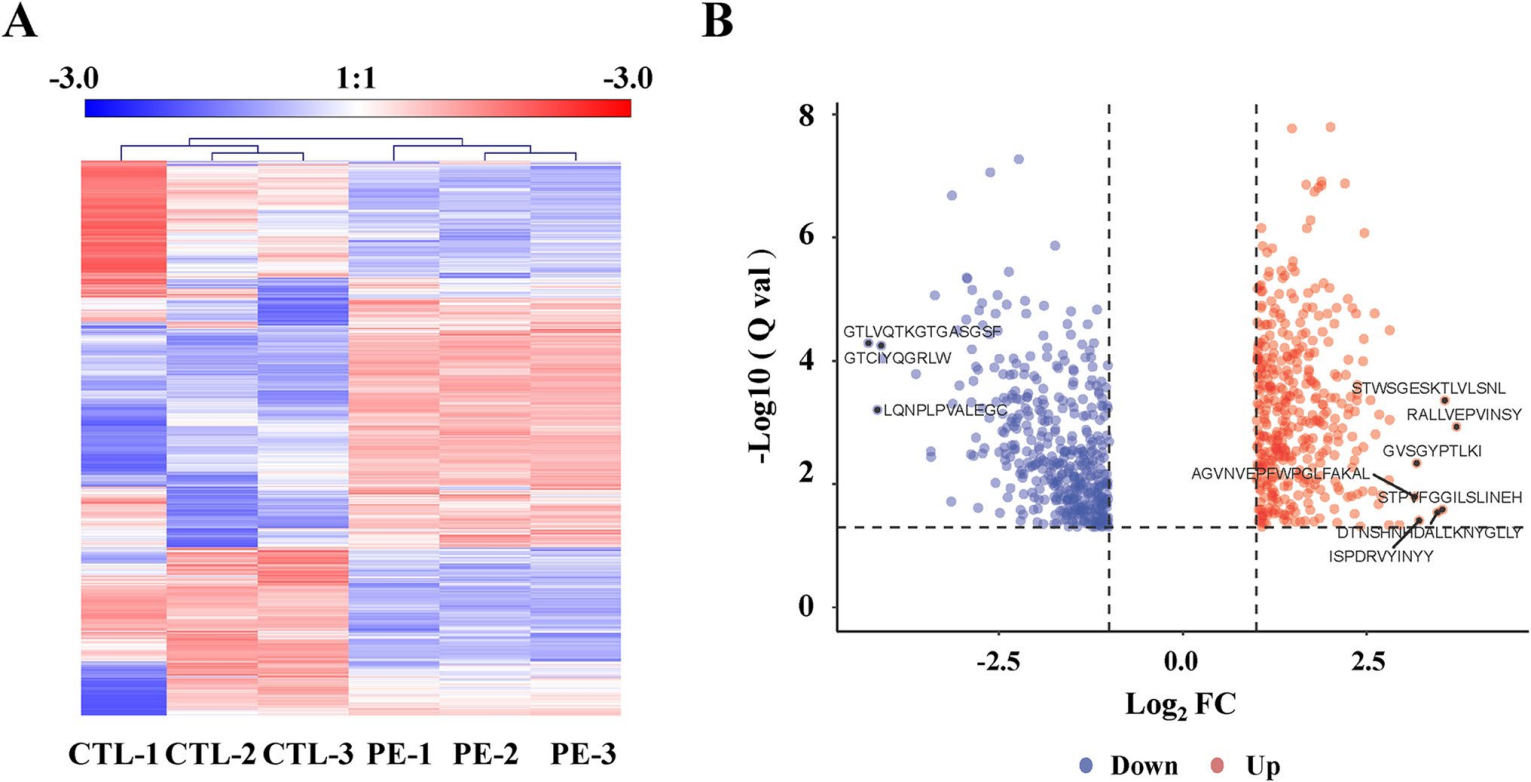
Differentially expressed peptides in healthy pregnant women and pregnant women with PE. The heatmap (**A**) and volcano plot (**B**) for the 812 peptides (absolute fold change ≥ 2 and adjusted *P* value < 0.05).

After labeling, peptides in the human placenta from women with PE and healthy groups were directly analyzed by LC–MS/MS. A total of 3494 peptides were identified from these two groups. After using BH methods to perform multiple test calibrations, among them, 812 were found to be differentially expressed (adjusted *P* value < 0.05, absolute fold change ≥ 2). Specifically, 420 were downregulated and 392 were upregulated. Clustering analysis (heatmap) and volcano plot of the 812 peptides are shown in Fig. 1. In addition, we have listed the sequence of the top 10 differentially expressed peptides in Table 2 and labeled these peptides in Fig. 1B. These peptides were mapped to 9 protein precursors, 7 of these peptides presented high levels (a fold change > 3.0, *P* < 0.05) in placenta from PE patients, while 3 peptides accounted for the main composition of peptides in placenta from women of normal pregnancy.

**Table 2:** Top 10 differentially expressed peptides between PE and normal pregnancies.

Parent protein	Peptide	Fold change	*P* value	Q-value
Transglutaminase 2 (TGM2)	RALLVEPVINSY	4.27	< 0.001	0.001
Nucleolin (NCL)	STWSGESKTLVLSNL	4.11	< 0.001	< 0.001
Tripeptidyl peptidase 1 (TPP1)	STPVFGGILSLINEH	4.08	0.011	0.026
Chorionic somatomammotropin hormone 1 (CSH1)	DTNSHNHDALLKNYGLLY	4.01	0.012	0.029
Macrophage migration inhibitory factor (MIF)	ISPDRVYINYY	3.76	0.018	0.039
Protein disulfide-isomerase A3 (PDIA3)	GVSGYPTLKI	3.73	0.001	0.005
Acidic ribosomal protein P1 (RPLP)	AGVNVEPFWPGLFAKAL	3.71	0.006	0.016
Histone H1.2 (HIST1H1C)	GTLVQTKGTGASGSF	− 3.72	< 0.001	< 0.001
Transglutaminase 2 (TGM2)	LQNPLPVALEGC	− 3.60	< 0.001	0.001
Neutrophil defensin 1 (DEFA1)	GTLVQTKGTGASGSF	− 3.54	< 0.001	< 0.001

### Details and cleavage sites of endogenous peptides

**Figure 2:**
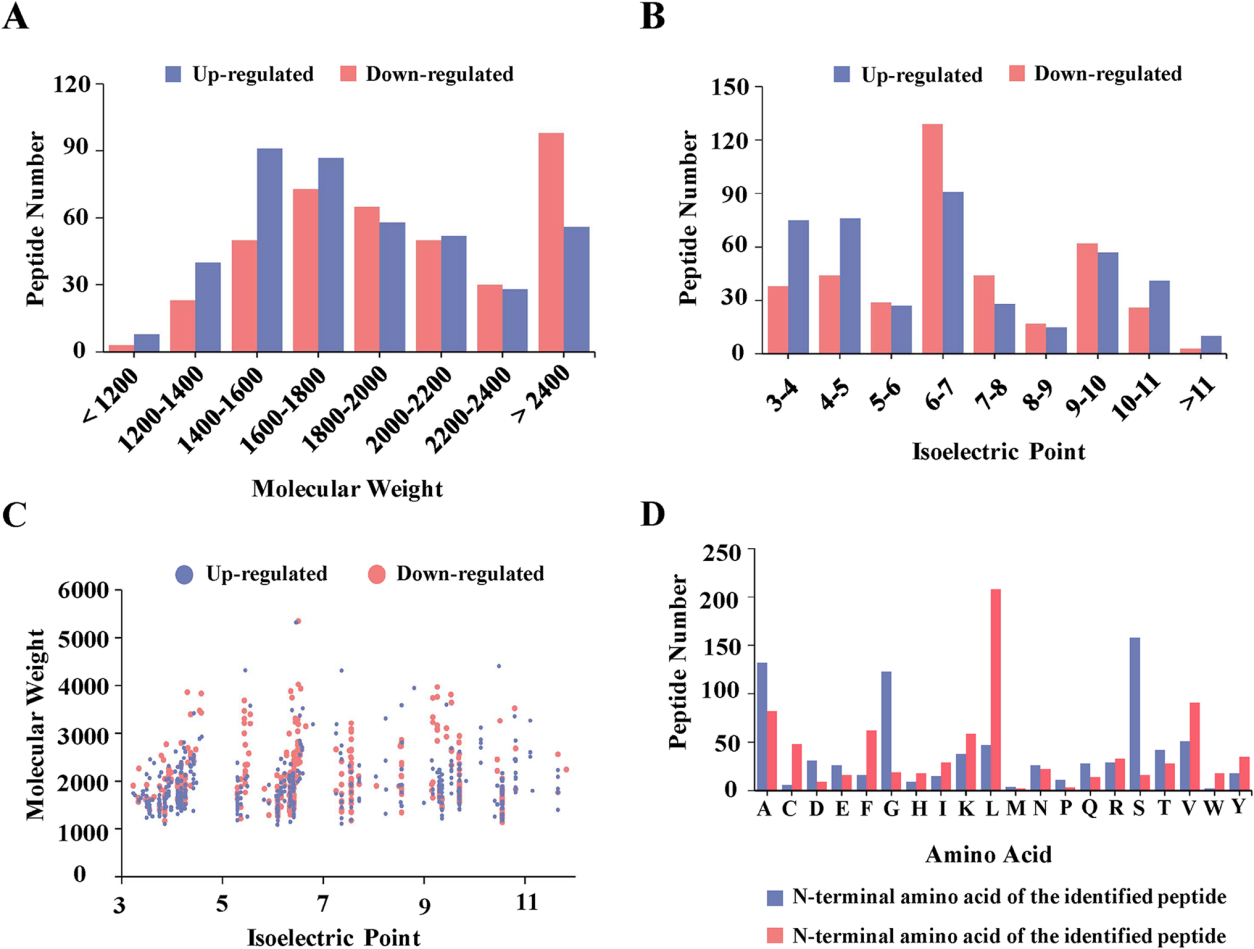
Features of the differentially expressed peptides. (**A**) Molecular weights (MWs) of the peptides. (**B**) Isoelectric points (pIs) of the peptides. (**C**) Scatter plot for the MWs versus the pIs. (**D**) Distributions of N-terminal (i.e. left-side) and C-terminal (i.e. right-side) amino acids of the identified peptides.

MW and pI were used to analyze the broad features of the differentially expressed peptides from PE and normal groups. The MW of all differential peptides ranged from 1090–5400 Da and the pI distribution ranged from 3 to 12 (Fig. 2A,B). In addition, we also studied the distribution of the MW relative to the pI (Fig. 2C). To further investigate the differentially expressed peptides, bioinformatics analyses were used to determine the amino acids at the cleavage sites of both the N- and C-termini. The results showed that glycine (G), alanine (A) and serine (S) dominated the cleavage sites at the N termini, while leucine (L), alanine (A) and valine (V) accounted for the most popular cleavage sites at the C terminal (Fig. 2D).

### Functional prediction of peptides based on precursor proteins

In order to evaluate the underlying biological functions of the differentially expressed peptides, we chose to study the precursor proteins of these peptides. GO analysis was used to determine the main biological functions of the precursor proteins and pathway analysis was used to identify the most important metabolic pathways and signal transduction pathways that these precursor proteins were involved in. Moreover, IPA software was also used to comprehensively analyze these precursor proteins.

**Figure 3:**
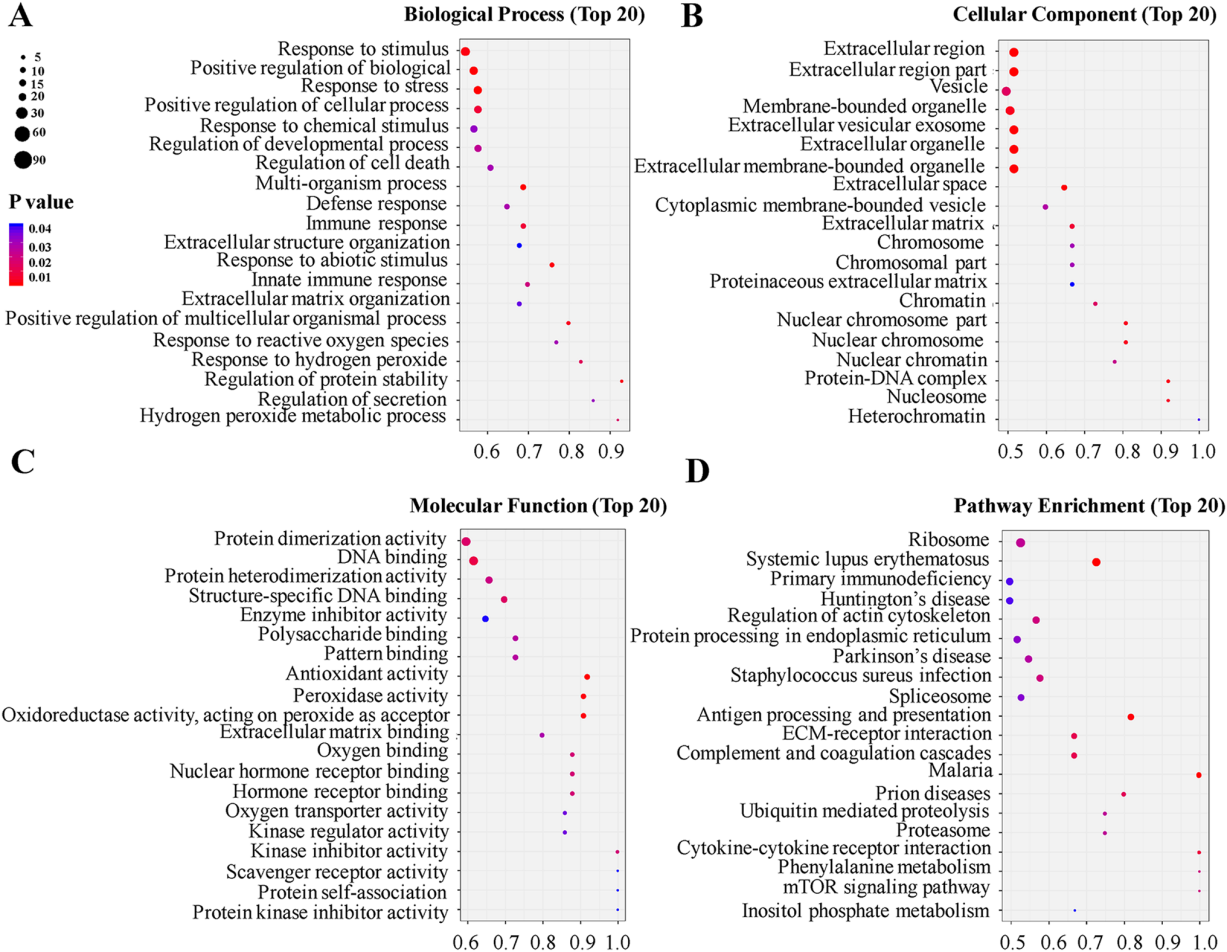
Gene ontology and pathway analysis of the precursor proteins of the differentially expressed peptides. (**A**) The biological process categories. (**B**) The cellular component categories. (**C**) The molecular function categories. (**D**) Canonical signaling pathways^43^.

GO analysis identified the response to stimulus, positive regulation of biological process, response to stress, positive regulation of cellular process, and response to chemical stimulus as the top 5 biological processes related to the differentially expressed peptides identified (Fig. 3A). Regarding cellular components, the top 5 highly enriched subcategories were extracellular region, extracellular region part, vesicle, membrane-bounded vesicle, and extracellular vesicular exosome. (Fig. 3B) For molecular functions, protein dimerization activity, DNA binding, protein heterodimerization activity, structure-specific DNA binding and enzyme inhibitor activity were the top 5 highly enriched subcategories (Fig. 3C). In addition, pathway analysis showed that the top 5 networks of these peptide precursors were ribosome, systemic lupus erythematosus, primary immunodeficiency, Huntington’s disease and regulation of actin cytoskeleton (Fig. 3D).

**Figure 4:**
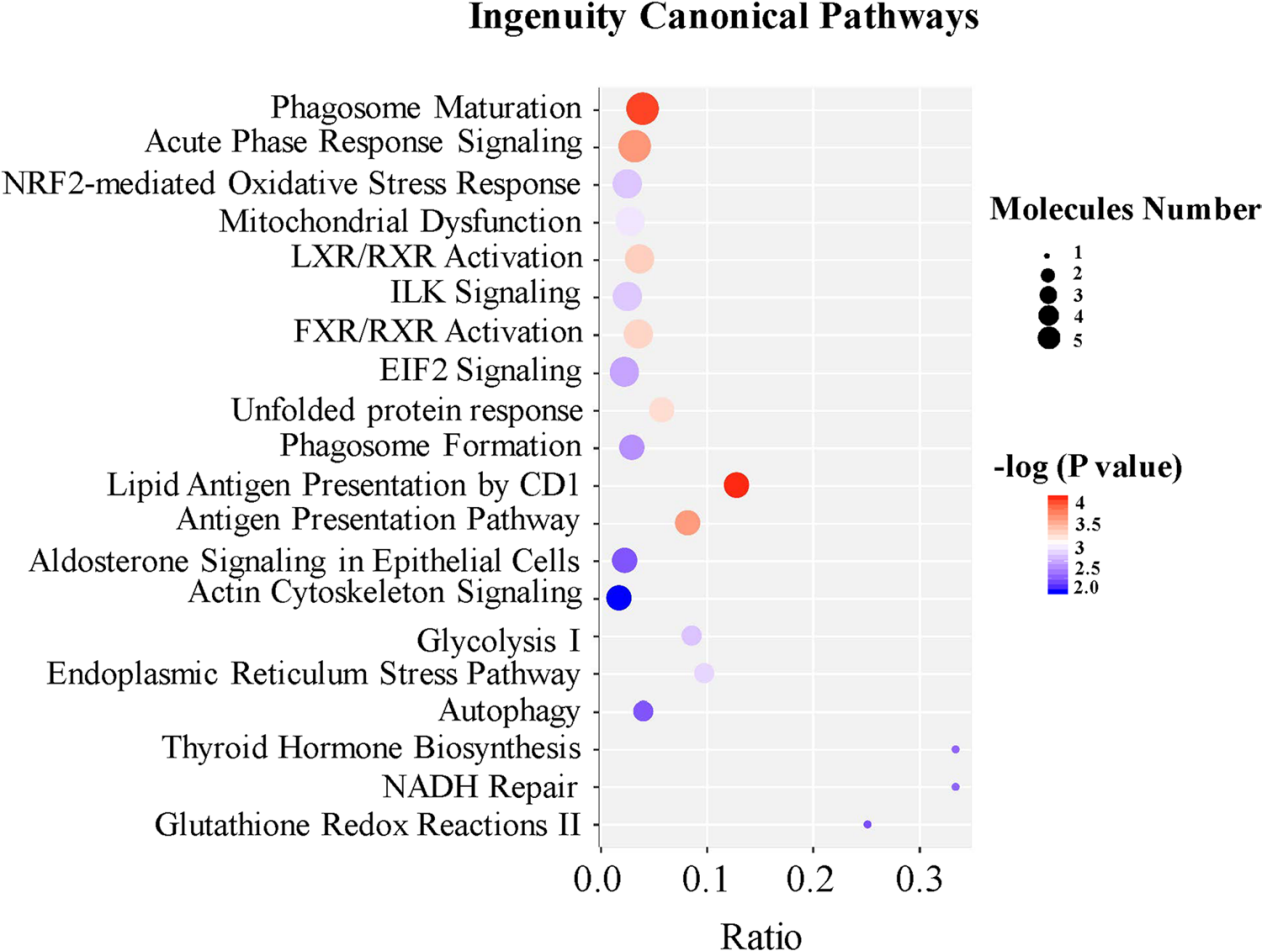
Pathway of the peptide precursors.

**Figure 5:**
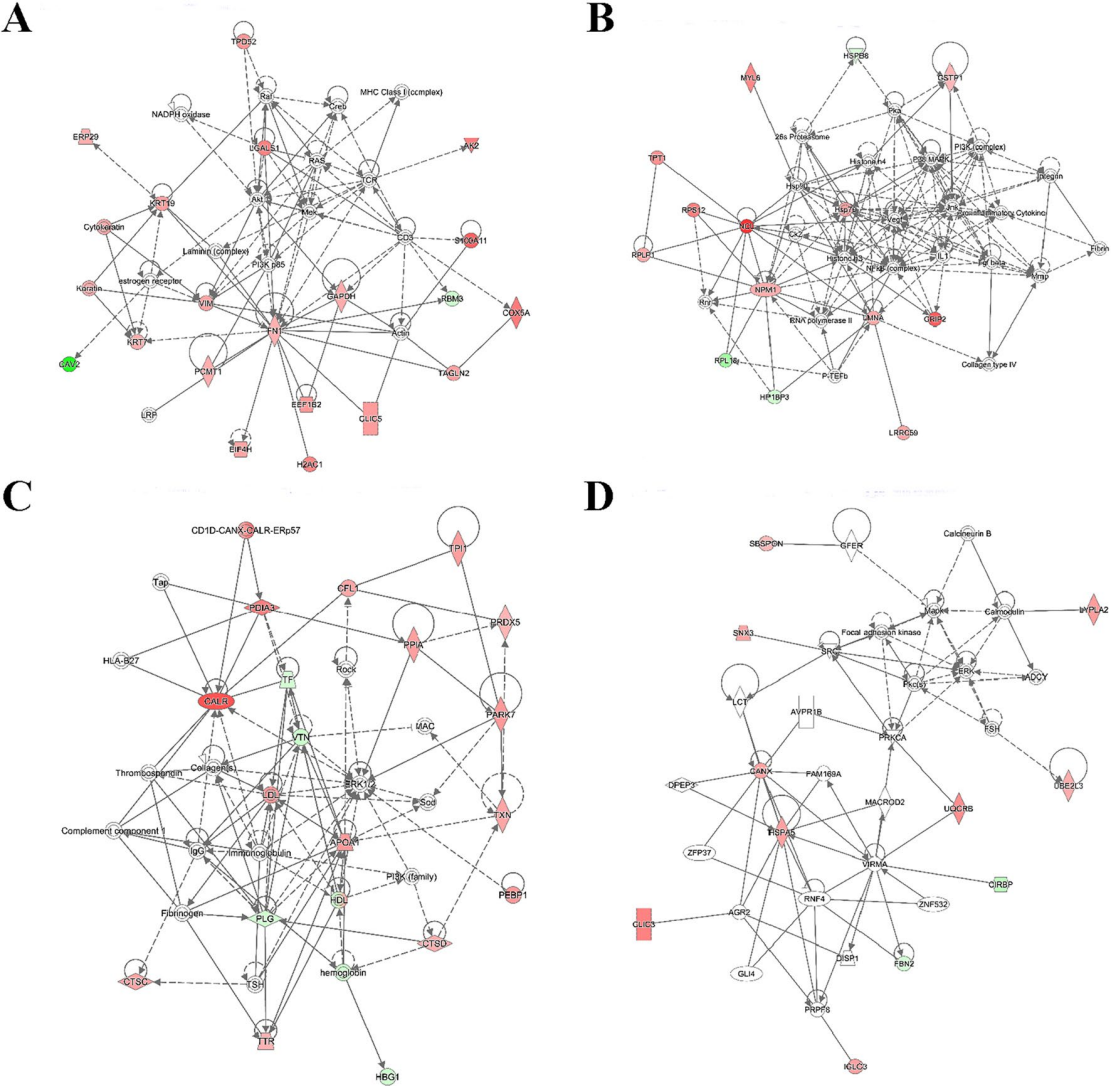
Interaction network of the peptide precursors. Protein network interaction map. The interaction line between orange and blue indicates the predicted activity state; orange indicates activation, blue indicates inhibition, and yellow indicates that the activity status of the target gene network regulated by the two regulators is inconsistent. The gray line indicates that the relationship is not quantified. Experimental validation or a clear quantitative relationship between protein–protein interactions.

IPA was performed to reveal the pathway of the protein precursors and the interaction network of genes related to differentially expressed peptides^23^. The top 5 of canonical pathway analysis are phagosome maturation, acute phase response signaling, NRF2-mediated oxidative stress response, mitochondrial dysfunction and LXR/RXR activation (Fig. 4, adjusted *P* value < 0.05 and absolute fold change ≥ 2). We also analyzed the interaction between genes by using network diagram (Fig. 5). For example, FN1 may regulate cell adhesion and migration by regulating Pcmt1, eif4h, h2ac1, eef1b2, clic5, Tagln2, RBM3 and other genes to affect the occurrence of preeclampsia (Fig. 5A). In addition, we found that most genes are related to metabolic diseases. In addition, we found that many genes are related to metabolic diseases, such as AKT was involved in glucose metabolism, APOA1 was related to lipid metabolism, and HP1BP3 seem to be associated with thyroid disease.

### Effect of identified bioactive peptides on the function of HTR-8/SVneo

Firstly, according to the expression difference, we chose three most significant peptides “RALLVEPVINSY” derived from Transglutaminase 2 (TGM2), “STWSGESKTLVLSNL” derived from Nucleolin (NCL) and “STPVFGGILSLINEH” derived from Tripeptidyl peptidase 1 (TPP1) to further explore their potential function (Table 1). Considering that the invasion of trophoblasts is essential for establishing uteroplacental circulation; poor invasion of trophoblasts can impair the remodeling of the spiral arteries, which results in maternal endothelial cell dysfunction, and ultimately leads to hypoperfusion and clinical manifestations of PE^24^. Therefore, we determined the invasive function of these peptides on HTR-8 cells, but unfortunately the experimental results did not show significant differences (Supplemental Fig. S1).

**Figure 6:**
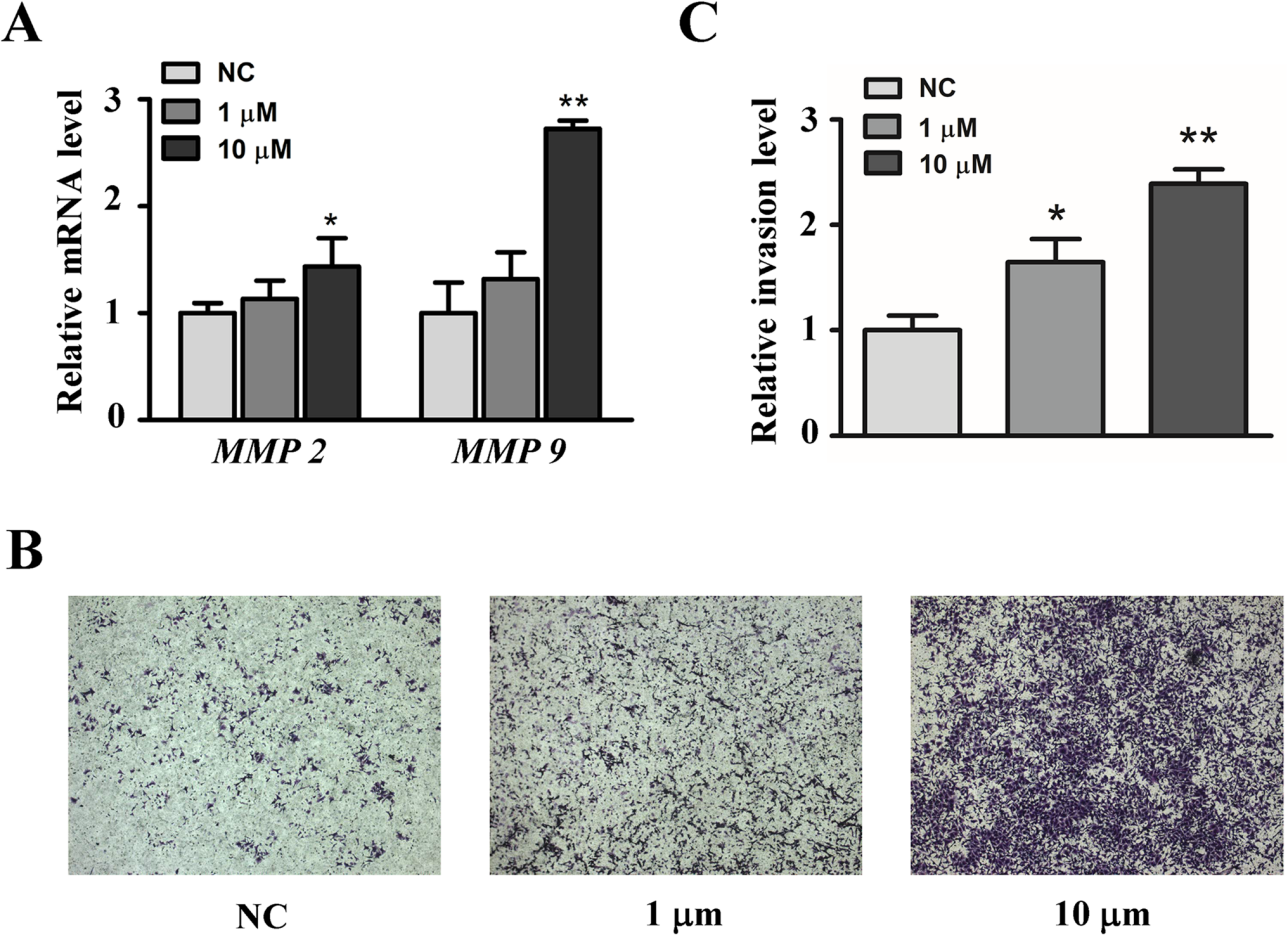
Function analysis of the peptide “DQSATALHFLGRVANPLSTA.” (**A**) Quantitative PCR, (**B**,**C**) Transwell assay. *NC* negative control.

It has been reported that the endogenous peptides may exhibit identical or opposite effects with their precursor proteins^25^. Besides hypertension and proteinuria, preeclampsia is characterized by a high peripheral vascular resistance and a reduced intravascular volume. Mating of female transgenic mice expressing angiotensinogen with males expressing renin leads to a preeclampsia-like phenotype with hypertension, proteinuria and kidney injury. Notably, AGT also shows significant effect on the invasion function of the trophoblasts. So, we selected two peptides “DQSATALHFLGRVANPLSTA” and “ATALHFLGRVANPLSTA” derived from AGT to evaluate their effects on invasion activity of HTR-8/SVneo cells (Supplemental Table S2). qPCR results indicated that the peptide “DQSATALHFLGRVANPLSTA” enhanced the relative mRNA expression of Matrix metalloproteinases-2 (MMP-2) and matrix metalloproteinases-9 (MMP-9) (Fig. 6A). MMP-2 and MMP-9 were widely known to be associated with cell invasion. Moreover, we used Transwell analysis to examine whether the peptide affects the invasion function of HTR-8/SVneo cells. As shown in Fig. 6B,C, the peptide significantly enhanced the invasive function of HTR-8, especially when the concentration reached 10 μM.

## Discussion

The exact mechanisms underlying the development of PE are still elusive, the eventual way to cure PE is to deliver the placenta and baby, which often results in preterm birth^26^. Thus, placenta has historically considered as the origin of the disease. Although once misunderstood as inactive intermediates from the protein degradation, intracellular peptides recently exhibit multiple functions within cells, including mitochondria protein stability, glucose metabolism, signal transduction as well as calcium level maintenance^27–31^. Thus, we performed a LC–MS/MS analysis to dissecting endogenous peptides profile in placenta, hoping to find some new ideas for PE treatment.

Currently, with the rapid development of peptide technology, hundreds of peptides derived from proteins have been identified and found involving in the pathogenesis process of diseases. For example, ELA, a peptidic hormone enriched in placenta, works as an endogenous ligand for the apelin receptor. Administration of synthesized ELA to pregnant ELA-deficient mice with a preeclamptic phenotype could ameliorate proteinuria and kidney injury without affecting embryonic development^32^. However, the number of endogenous peptides is hug, a comprehensive profiling of placental endogenous peptides is necessary. Presently, we mapped the intracellular peptide profile of placenta, and a total of 3492 peptides were identified. Of these peptides, 812 peptides were found to be differentially expressed. These expression alterations of peptides between two groups shed insight relevance to the pathogenesis of PE.

The regulation of proteases results in the distinct production of peptides and may contribute to the disease processes^25^. Therefore, understanding the substrate cleavage sites is helpful to interpret the host response to physiological states. We determined the amino acids at the cleavage sites of both the N- and C-termini by statistical locus analysis. We can see that L was the most hit cleavage site for C-terminal amino acid of the identified peptide, while S dominated the cleavage site of N-terminal amino acid of the identified peptide. This analysis provided a global view of placental protease combined with site-specific protease cleavage events, and also suggests that the activity of proteases is regulated by the pathological processes of PE.

PE is a complicated pathological process related to a maternal milieu that is predisposed or susceptible to hypertension^33^. The GO analysis revealed that the biological processes of the differentially expressed peptides were associated with response to reactive oxygen species (ROS). In addition, it is also related to the regulation of blood glucose level, blood lipid levels, bile metabolism, and thyroid function^34–37^. The pathogenesis of PE is still not very clear, but most researchers believe that oxidative stress plays an important role. The ROS produced by oxidative stress will damage trophoblast cells and release many pro-inflammatory factors. In addition, elevated ROS levels are clearly found in the placentae of pregnant women with PE^38^. To the best of our knowledge, the VEGF signaling pathway is highly related to the function of placental trophoblast cells. Reduced levels of VEGF may affect the invasiveness of trophoblasts, leading to defects in trophoblast function and affecting the revascularization of blood vessels, which may eventually cause PE^39^. These results indicate that these differential peptides may be involved in the development of PE and play an important regulatory role.

The invasiveness of trophoblast cells is due to their ability to secrete MMPs, which play a key role in uterine spiral arterial recasting and placenta formation, and MMP-2 and MMP-9 are the two most important proteases. The increase of MMP-2 and MMP-9 is important for maintain normal pregnancy, which can affect the expansion of vasodilation, placentation, and uterine expansion. The abnormal expression of MMP-2 and MMP-9 in trophoblast cells may be an important cause of PE^40,41^. Experiment in our study confirmed that AGT derived peptide “DQSATALHFLGRVANPLSTA” increased the expression of MMP-2 and MMP-9 in HTR-8/SVneo cells. In addition, the invasion assay also showed that this peptide can increase the invasion of trophoblast cells. The hypothesis that trophoblastic infiltration defects caused by hypoperfusion of the uterus and placenta can lead to PE has been supported by previous studies^42^. Therefore, we boldly speculate that the secretion of this peptide may be related to the pathogenesis of PE, and perhaps after further research, it may become a new lead for preventing, diagnosing, or treating PE.

## Conclusion

Our study is the first report to compare the peptide profiles of placentae from healthy pregnant women and pregnant women with PE for a deeper understanding of PE from a peptidomic perspective. These findings bring new prospects for the prediction, diagnosis, and treatment of PE, and provide a new perspective for further research on the mechanism underlying PE. However, some limitations should be noted in present study. First, the count of samples in this study is low, more participants is needed in future study. Second, a deeper exploration is lacking to find more functional peptides, science these peptides may work in different ways to drive the PE process. Third, the effect of peptide “DQSATALHFLGRVANPLSTA” derived from AGT in PE treatment must be varied via animal experiment.

## Supplementary information

Supplementary Information

## Data Availability

The mass spectrometry proteomics data have been deposited to the ProteomeXchange Consortium (https://proteomecentral.proteomexchange.org) via the iProX partner repository with the dataset identifier PXD016030.

## Abbreviations

ACOG: American College of Obstetricians and Gynecologists
BH procedure: Benjamini–Hochberg procedure
BMI: Body mass index
FBS: Fetal bovine serum
FDR: False discovery rate
GO: Gene ontology
IPA: Ingenuity pathway analysis
LC–MS/MS: Liquid chromatography–tandem mass spectrometry
MMP: Matrix metalloproteinases
MW: Molecular weight
PE: Preeclampsia
pI: Isoelectric point
qPCR: Quantitative PCR
TMT: Tandem mass tag
