# Supplementary material for: Distinct expression profiles of peptides in placentae from preeclampsia and normal pregnancies
Source: Sci Rep. 2020 Oct 16;10:17558. doi: 10.1038/s41598-020-74840-0 (PMC7567870; doi:10.1038/s41598-020-74840-0)
Supplement: Supplementary file 1 — Supplementary Information [file 41598_2020_74840_MOESM1_ESM.docx]

**Distinct Expression Profiles of Peptides in Placentae from Preeclampsia and Normal Pregnancies**

Jin Huang^1,2,*^ , Zhonghui Ling^1,*^, Hong Zhong^3^, Yadong Yin^1^, Yating Qian^1^, Mingming Gao^1,3^, Hongjuan Ding^1^, Qing Cheng^1,#^, Ruizhe Jia^1,#^

*^1^Women's Hospital of Nanjing Medical University, Nanjing Maternity and Child Health Care Hospital, Nanjing, Jiangsu 210004, China*

*^2^Yixing People’s Hospital, YiXing Jiangsu 214200, China*

*^3^Fourth Clinical Medicine College, Nanjing Medical University, Nanjing Jiangsu 210000, China*

*These authors contributed equally to this work

**#Address Correspondence to Ruizhe Jia:**

Women’s Hospital of Nanjing Medical University, Nanjing Maternity and Child Health Care Hospital, Nanjing, 210004, China.

Tel: +86 25 52226159; E-mail address: jiaruizhe2016@163.com

**#Address Correspondence to Qing Cheng:**

Women’s Hospital of Nanjing Medical University, Nanjing Maternity and Child Health Care Hospital, Nanjing, 210004, China.

Tel: +86 25 52226162; E-mail address: qqingrr@163.com

**Supplemental Materials**


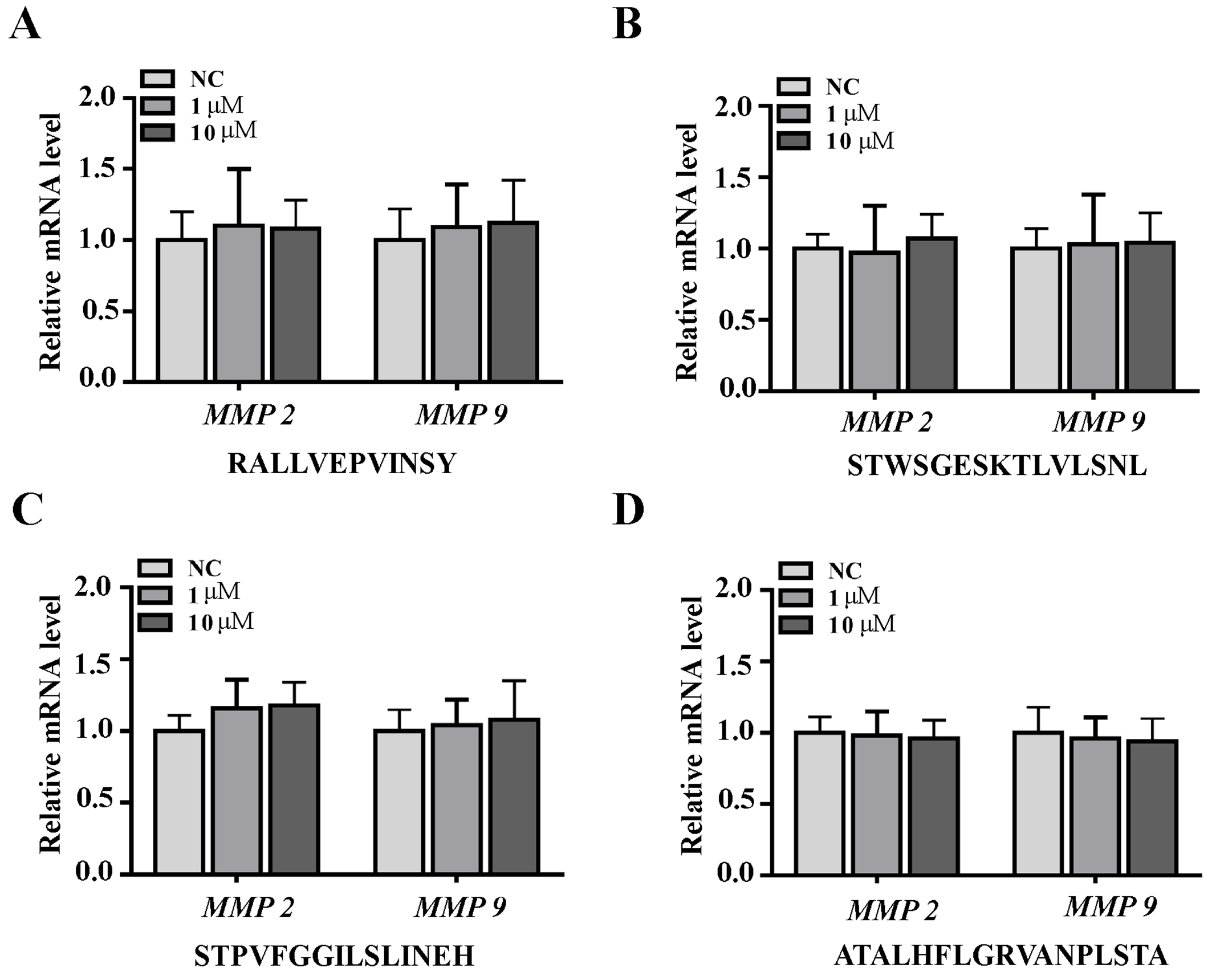


**Supplemental Figure S1 Relative expression levels of MMP 2 and MMP 9 in HTR-8/SVneo cells treated with different peptides.** Cells were separately treated with peptides derived from Transglutaminase 2 (A), Nucleolin (B), Tripeptidyl peptidase 1 (C) and Angiotensinogen (D).

**Supplemental Table S1. Sequence of primers used in our study**

|  | 5’-3’ (Sense) | 5’-3’ (Antisense) |
| --- | --- | --- |
| Homo-MMP-2 | CCCCAGACAGGTGATCTTGAC | GCTTGCGAGGGAAGAAGTTG |
| Homo-MMP-9 | GGTTCAGGGCGAGGACCATAGAG | TTTGACAGCGACAAGAAGTGGG |
| Homo-GAPDH | GGAGCGAGATCCCTCCAAAAT | GGCTGTTGTCATACTTCTCATGG |

**Supplemental Table S2. Peptides derived from ANGT**

| **Parent protein** | **Peptide** | **Fold change** | **P-value** | **Q-value** |
| --- | --- | --- | --- | --- |
| ANGT | DQSATALHFLGRVANPLSTA | 1.82 | < 0.001 | 0.003 |
| ANGT | ATALHFLGRVANPLSTA | -0.85 | 0.001 | 0.004 |
